# Serum micro-RNAs with mutation-targeted RNA modification: a potent cancer detection tool constructed using an optimized machine learning workflow

**DOI:** 10.1038/s41598-024-59480-y

**Published:** 2024-04-19

**Authors:** Wei Liao, Yuyan Xu, Mingxin Pan, Huanwei Chen

**Affiliations:** 1 Department of Hepatobiliary Surgery, The First People’s Hospital of Foshan, Foshan, Guangdong Province China; 2 General Surgery Center, Department of Hepatobiliary Surgery II, Zhujiang Hospital, Southern Medical University, Guangzhou, Guangdong Province China

**Keywords:** microRNAs, RNA modification, Single-nucleotide variants, Cancer detection, Machine learning, Cancer, Computational biology and bioinformatics, Biomarkers

## Abstract

RNA modifications affect fundamental biological processes and diseases and are a research hotspot. Several micro-RNAs (miRNAs) exhibit genetic variant-targeted RNA modifications that can greatly alter their biofunctions and influence their effect on cancer. Therefore, the potential role of these miRNAs in cancer can be implicated in new prevention and treatment strategies. In this study, we determined whether RMvar-related miRNAs were closely associated with tumorigenesis and identified cancer-specific signatures based on these miRNAs with variants targeting RNA modifications using an optimized machine learning workflow. An effective machine learning workflow, combining least absolute shrinkage and selection operator analyses, recursive feature elimination, and nine types of machine learning algorithms, was used to screen candidate miRNAs from 504 serum RMvar-related miRNAs and construct a diagnostic signature for cancer detection based on 43,047 clinical samples (with an area under the curve value of 0.998, specificity of 93.1%, and sensitivity of 99.3% in the validation cohort). This signature demonstrated a satisfactory diagnostic performance for certain cancers and different conditions, including distinguishing early-stage tumors. Our study revealed the close relationship between RMvar-related miRNAs and tumors and proposed an effective cancer screening tool.

## Introduction

Cancer is a leading cause of death and the single most stringent barrier to increasing life expectancy worldwide^1^. According to the recent statistics from GLOBCAN, a total of 19.3 million new cancer cases, with approximately 10.0 million cancer-related deaths, occurred globally in 2020^2^, thereby considerably hindering socioeconomic development. As cancer often present no obvious symptoms during the early stage, most patients are diagnosed with cancer at an advanced stage, leading to higher treatment costs, a greater possibility of metastasis, and increased mortality.

The World Health Organization has indicated that early detection is the most effective strategy for managing cancer and increasing survival rate by potentially providing patients with rapid treatment in the early stages^3^. Therefore, developing a cost-effective and accurate cancer detection tools will improve individual life expectancy and decrease socioeconomic hardship.

MicroRNAs (miRNAs) are noncoding RNAs with a length of 19–25 nucleotides associated with numerous biological processes, such as cell differentiation, proliferation, apoptosis, and inflammation^4^. miRNAs, discharged into serum by cells through exosomes, reflect the specific molecular signatures of that cell and exist stably in the blood^5^. Aberrant expressions of miRNAs have been observed in lung^6^ and breast cancers^7^, hepatocellular carcinoma (HCC)^8^, prostate cancer^9^, oral squamous cell carcinoma^10^, and colorectal cancer^11^. These tumor-specific miRNAs can be detected in the blood via tumor-derived exosomes. Exosomal miRNAs may serve as biomarkers that provide stable, sensitive, and precise biological information^12^. Therefore, identifying biomarkers of serum miRNAs with high efficacy is a promising approach to facilitate non-invasive diagnosis of cancer prior to clinical manifestation and to lower cancer-related mortality.

Single-nucleotide variants (SNVs) are the most common genetic variants and are universally present in the human genome. Numerous disease- or trait-associated variants have been identified by genome-wide association studies^13^. Variants can impact protein coding, influence RNA–protein interactions, alter the secondary structure of RNA, and change the splicing sites of RNA^14^. The genetic variants specifically substituting the nucleotide at the modified position or changing the nucleotide sequence in the proximal flanking region can influence RNA modifications^15^. RNA modifications are critical posttranscriptional regulators of gene expression programs, as they are crucial for modulating coding and non-coding RNA metabolism and affect diverse eukaryotic biological processes^16^. Alterations in their deposition are implicated in several diseases and cancers. For example, the abnormal N6-methyladenosine (m6A) methylation, one of the most common RNA modifications, is involved in various cancers, including leukemia, glioblastoma, lung cancer, liver cancer, and bladder cancer, by regulating cell proliferation, metastasis, stem cell differentiation, and homeostasis^17^.

Various miRNAs contain variants targeting RNA modifications that may alter their biofunctions and expression^18^. However, the clinical implications and biofunctions of these miRNAs with variants targeting RNA modifications remain largely unknown.

Therefore, in this study, we comprehensively analyzed the expression feature of 504 serum miRNAs with variants targeting nine common RNA modifications, namely m6A, N6-dimethyladenosine (m6Am), N1-methyladenosine (m1A), pseudouridine (ψ), 5-methylcytosine (m5C), ribose methylations (2′-O-Me), 7-methylguanosine (m7G), 5-methyluridine (m5U), and adenosine-to-inosine (A-to-I), from 43,047 cancer and non-cancer controls. We used an optimized machine learning workflow to establish cancer-specific signatures based on these miRNAs with variants targeting RNA modifications.

## Materials and Methods

### Data collection and preprocessing

A pan-cancer-focused serum miRNA expression microarray was retrieved from the Gene Expression Omnibus. The normalized matrix files and corresponding clinical information were directly downloaded. Patients with a definitive diagnosis were first enrolled in our study, and then those who had received any anti-tumor treatment, including intervention, chemotherapy, radiotherapy, and targeted and immune therapies or surgery before serum collection, were screened out. Eleven eligible serum miRNA cohorts were ultimately included in the study, of which nine miRNA cohorts, including GSE106817, GSE112264, GSE113468, GSE113740, GSE122497, GSE124158, GSE137140, GSE139031, and GSE164174, were from the same platform as GPL21263 and subsequently integrated for model construction and validation. Microarray GSE73002, GSE59856, GSE85679, and GES124158 from platform GPL18941 and GSE211692 from GPL21263 were used for external validation. After eliminating duplicate samples, 23,026 control serum samples and 20,021 cancer serum samples were collected for further analysis. Our study involved 16 cancer types, including biliary tract cancer, bladder cancer, colorectal cancer, esophageal cancer, gastric cancer, glioma, HCC, lung cancer, malignant bone and soft tissue tumor, metastatic brain tumors, ovarian cancer, pancreatic cancer, primary central nervous system lymphoma, prostate cancer, sarcoma, and breast cancer, as well as non-cancer controls. The “ComBat” function of the sva package was utilized to perform a batch correction of different serum miRNA profiles^19^.

### Collection and function enrichment analysis of miRNAs with variants targeting RNA modification

RNA modifications can functionally regulate mRNA metabolism and affect several eukaryotic processes^16^. The proper deposition of RNA modifications is crucial for the physiological developmental processes. RNA modification variants are closely associated with dysregulation of cellular processes, resulting in serious diseases such as cancer^20^. Functional variants, particularly cancer mutations, can significantly alter RNA modification status, leading to the gain or loss of RNA modification sites^14^. We gathered miRNAs with variants targeting RNA modifications from the RMVar database, which consists of germline mutations from The Single Nucleotide Polymorphism Database, Human Genetic Variation Database, and somatic mutations from The Cancer Genome Atlas, International Cancer Genome Consortium, and Catalogue of Somatic Mutations in Cancer. It is also specifically designed to provide potential assistance in revealing the functional roles of RNA modification variants^18^. Notably, we extracted 504 miRNAs affected by several most common RNA modification variants (Supplemental Table 1), including m6A, m6Am, m1A, ψ, m5C, 2′-O-Me, m7G, m5U, and A-to-I. Differentially expressed miRNAs between patients with cancer and without cancer (controls) were detected using the “limma” package. Next, to investigate the biological processes influencing these miRNAs, we performed a functional enrichment analysis using the miRNA Enrichment Analysis and Annotation Tool^21^.

### Construction of diagnostic signature for pan-cancer detection through optimized machine learning workflow

**Figure 1:**
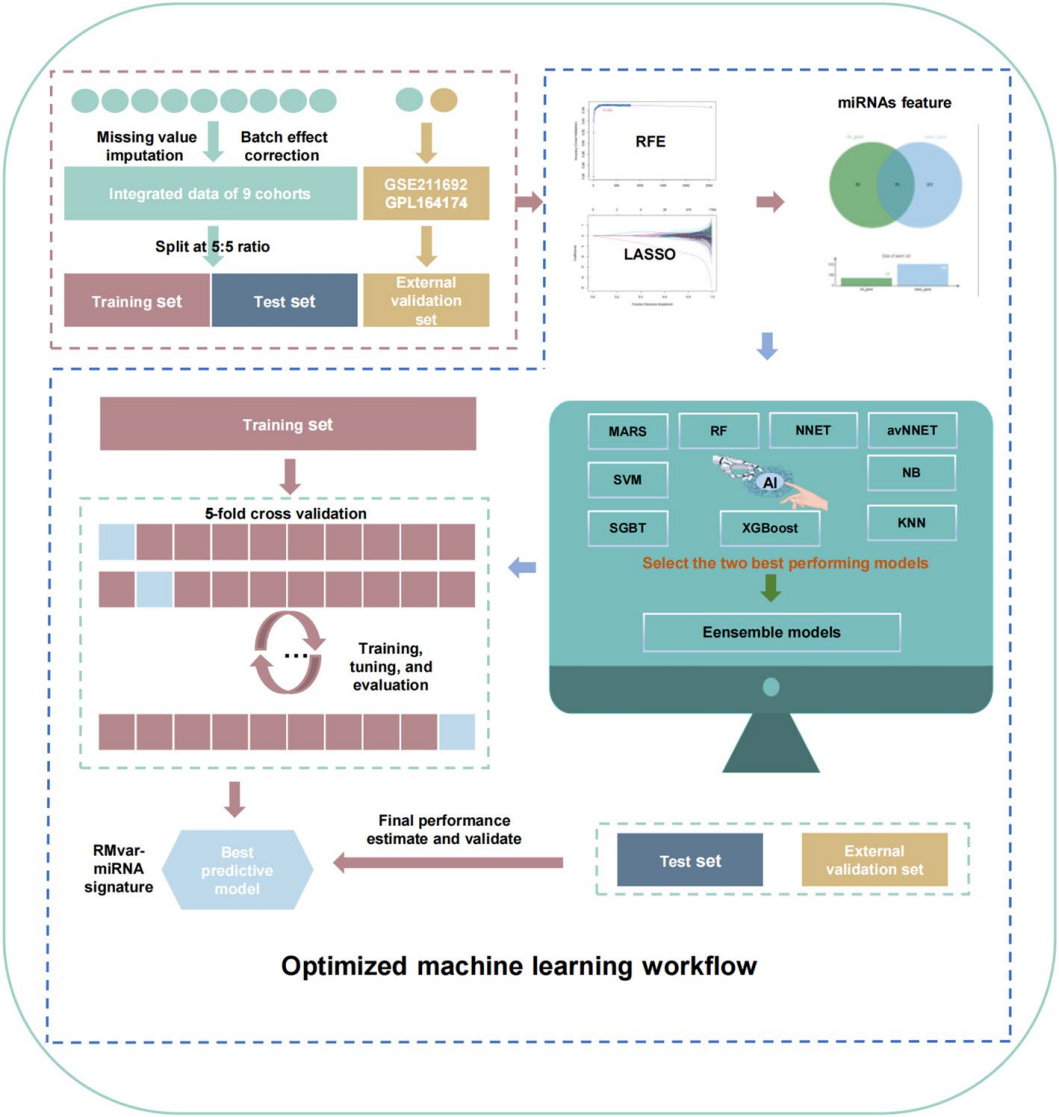
Optimized machine learning workflow used in the present study.

To establish the serum diagnostic signature for pan-cancer detection, we randomly divided serum samples from platform GPL21263 into training and validation cohorts at a ratio of 1:1 using the createDataPartition function of the “caret” R package^22^. Then, we applied the recursive feature elimination (RFE) and least absolute shrinkage and selection operator (LASSO) algorithms for feature selection in the training cohort. RFE and LASSO were performed using the “caret” and “glmnet” R packages, respectively. Next, the overlapping miRNAs between RFE and LASSO were extracted for further analysis, and 79 miRNAs were ultimately used to develop nine machine learning models, namely Multivariate Adaptive Regression Spline (MARS), Random Forest (RF), Neural Network (NNET), Model Averaged Neural Network (avNNET), Support Vector Machines (SVM) with Radial Basis Function Kernel, Stochastic Gradient Boosting Tree (SGBT), eXtreme Gradient Boosting (XGBoost), Naive Bayes (NB), and k-Nearest Neighbors (KNN). The “caret” R package was used to construct nine machine learning models with fivefold cross-validation for identifying the binary classification, which can optimize model performance and avoid overfitting. The “DALEX” package was applied to evaluate the model performance^23^. To improve the diagnostic model performance and increase the robustness and accuracy of the model, as shown by the machine learning community^24^, we constructed ensembles of the two best-performing models (SVM and SGBT) using the “caretEnsemble” R package. The diagnostic index, RMvar–miRNA signature, was calculated based on the predictive strength of the output of the ensembles from two machine learning classifiers. The “predict” function was applied to quantify the prediction signature score on the training and validation cohorts. The optimized machine-learning workflow used in the present study is illustrated in Fig. 1.

### Statistical analysis

Data processing was conducted using R (version 4.1.3) and the R Bioconductor package. The diagnostic performance of the RMvar–miRNAs signature was evaluated using receiver operating characteristic (ROC) curve analysis, including the area under the curve (AUC), sensitivity, specificity, and accuracy. The normality and homogeneity were tested using the Shapiro–Wilk normality test and Bartlett homogeneity test of variance, respectively. The nonparametric Wilcoxon and Kruskal–Wallis tests, parametric T-test, and one-way ANOVA were used to compare the differences. To investigate the function(s) of single nucleotide miRNAs and those of the m6A, m5C, and m7G mutations, we conducted an enrichment analysis based on KEGG and GO gene sets. All statistical analyses were two-sided, and *p* < 0.05 was considered statistically significant.

## Results

### Features and functional annotation of miRNAs with variants targeting RNA modifications

**Figure 2:**
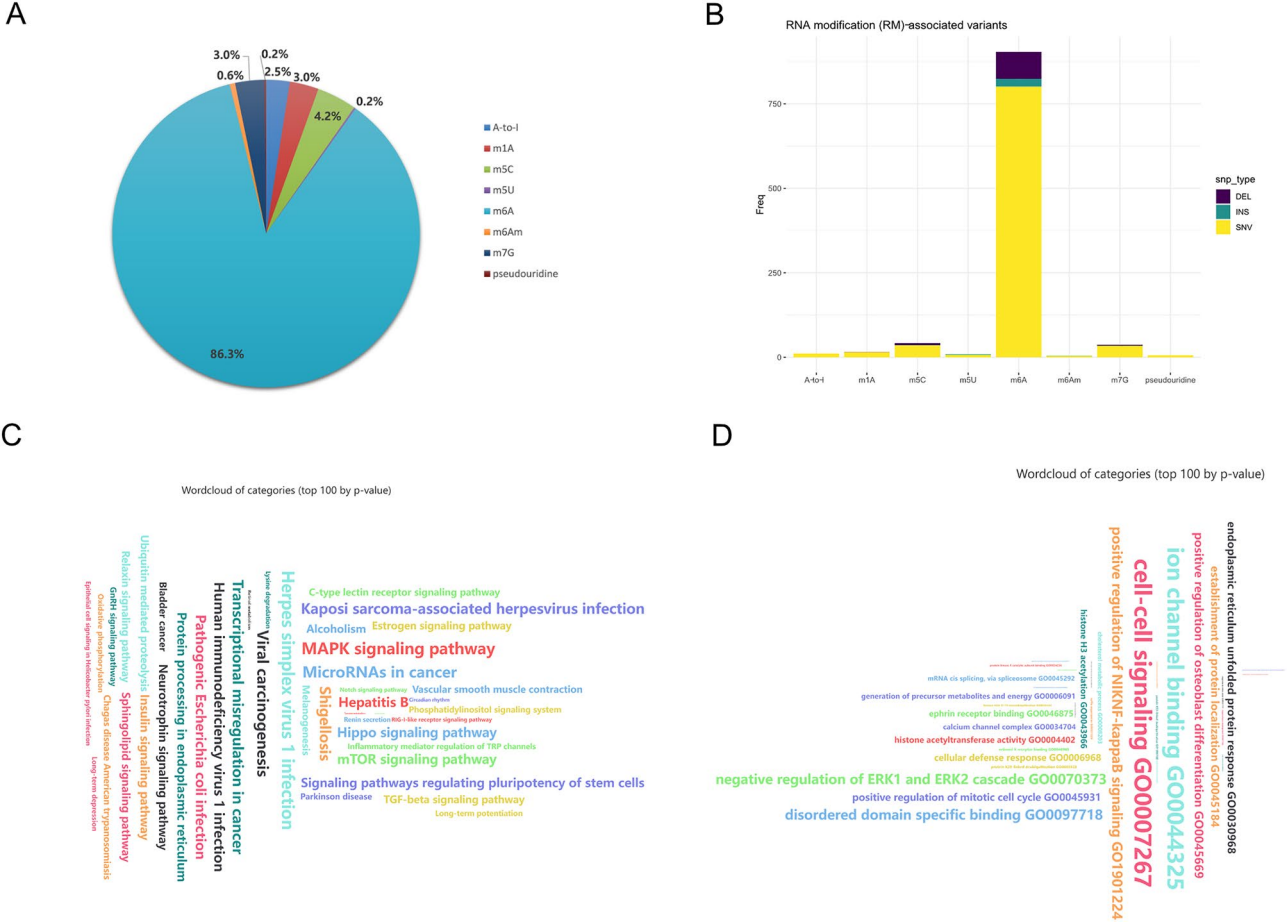
Features and functional annotation of miRNAs with variants targeting RNA modification. (**A**) Pie chart showing the proportions of the RNA modifications targeted by miRNA variants. (**B**) Types of single nucleotide polymorphism in the miRNA variants. DEL: deletion, INS: insertion, SNV: single nucleotide variant. (**C**) KEGG^26–28^ enrichment analysis of miRNAs with variants targeting RNA modifications (adjusted *p* < 0.05). The font size of the items represents the degree of enrichment. (**D**) GO enrichment analysis on miRNAs with variants targeting RNA modifications (adjusted *p* < 0.05). The font size of the items represents the degree of enrichment.

In this study, we collected 504 miRNAs with variants targeting RNA modifications, RMvar-related miRNAs, within the RMVar database (Supplementary Table 1). In those miRNAs, the m6A modifications accounted for the highest proportion (86.3%), followed by the m5C modification, with a proportion of 4.2% among the eight modifications mentioned previously. These findings were consistent with a previous study reporting that the m6A modification is the most common RNA modification in eukaryotes^25^ (Fig. 2A). As shown in Fig. 2B, most of the variants targeting RNA modifications had a single nucleotide, followed by a small amount of deletion mutation targeting the m6A, m5C, and m7G modifications. The KEGG enrichment analysis indicated that these miRNAs were associated with multiple pathways, including the MAPK signaling pathway, miRNAs in cancer, viral carcinogenesis, transcriptional misregulation in cancer, hepatitis B, and signaling pathways regulating pluripotency of stem cells (Fig. 2C, Supplementary Table 2). GO enrichment analysis demonstrated that these miRNAs were associated with cell–cell signaling, positive regulation of NIKNF-kappa B signaling, the endoplasmic reticulum-unfolded protein response, histone acetyltransferase activity, and generation of precursor metabolites and energy (Fig. 2D, Supplementary Table 3). These results imply that the RMvar-related miRNAs have broad biofunctions in cell regulation and are closely related to tumorigenesis.

### Screening of representative serum miRNAs and the construction of the cancer prediction model

**Figure 3:**
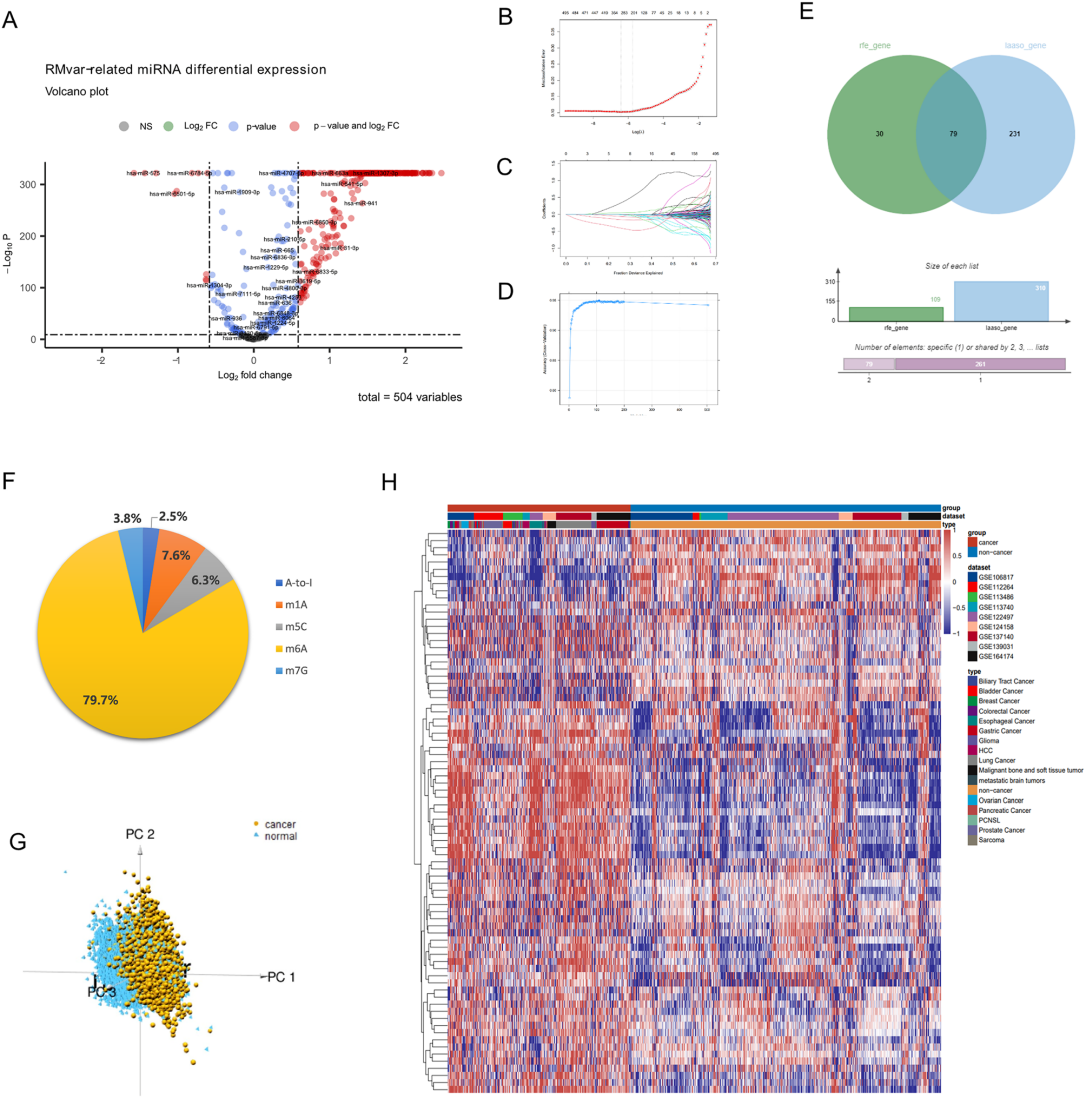
Screening of representative serum miRNAs from 504 RMvar-related miRNAs for constructing the cancer prediction model. (**A**) Volcano plot chart depicting the differentially expressed serum RMvar-related miRNAs in patients with and without cancer (|log2 fold change|> 1.5, adjusted *p* < 0.001). (**B**, **C**) Least absolute shrinkage and selection operator regression performed to screen the candidate NCOA6-related miRNAs based on the minimum criteria. (**D**) RFE algorithms in the tumor cohort. (**E**) Venn diagram depicting the 109 (screened using RFE, left circle) and 310 miRNAs with independent expression features (screened using LASSO analysis, right circle), as well as the 79 miRNAs in the intersection. (**F**) Pie chart showing the proportion of the RNA modification targeted by miRNA variants in the 79 representative serum miRNAs. (**G**) Principal component analysis (PCA) of 79 representative serum miRNAs on serum chip data from patients with and without cancer. (**H**) Unsupervised clustering analysis of 79 representative serum miRNAs on serum chip data from patients with and without cancer.

To investigate the clinical significance of the RMvar-related miRNAs, we first screened them for representative miRNAs. Figure 3A shows 298 serum RMvar-related miRNAs (in total 504 miRNAs) differentially expressed between patients with and without cancer, as screened using the “limma” package (|log2 fold change|> 1.5, adjusted *p* < 0.001) on the serum chip data. Notably, most of these miRNAs were highly expressed in cancers. The LASSO analyses and RFE were used to screen miRNAs with independent expression features in the 504 RMvar-related miRNAs based on the traing cohort.

Notably, 310 and 109 RMvar-related miRNAs were screened out (Fig. 3B–D, Supplementary Tables 4 and 5), and 79 RMvar-related miRNAs in the intersectional part of these two groups served as representative miRNAs for constructing the cancer prediction model (Fig. 3E, Supplementary Table 6). As shown in Fig. 3F, five types of RNA modifications—m6A, m5C, m1A, m7G, and A-to-I—were targeted by the variants of the 79 representative miRNAs. The m6A modification accounted for the largest proportion (79.7%), consistent with previous results. The principal component analysis (PCA) based on the 79 representative miRNAs demonstrated a significant difference between patients with and without cancer, confirming that the 79 miRNAs were suitable for constructing the cancer prediction model (Fig. 3G). Moreover, the unsupervised clustering analysis based on the serum chip data demonstrated that these 79 representative miRNAs were differentially expressed in patients with and without cancer, consistent with the PCA results (Fig. 3H).

**Figure 4:**
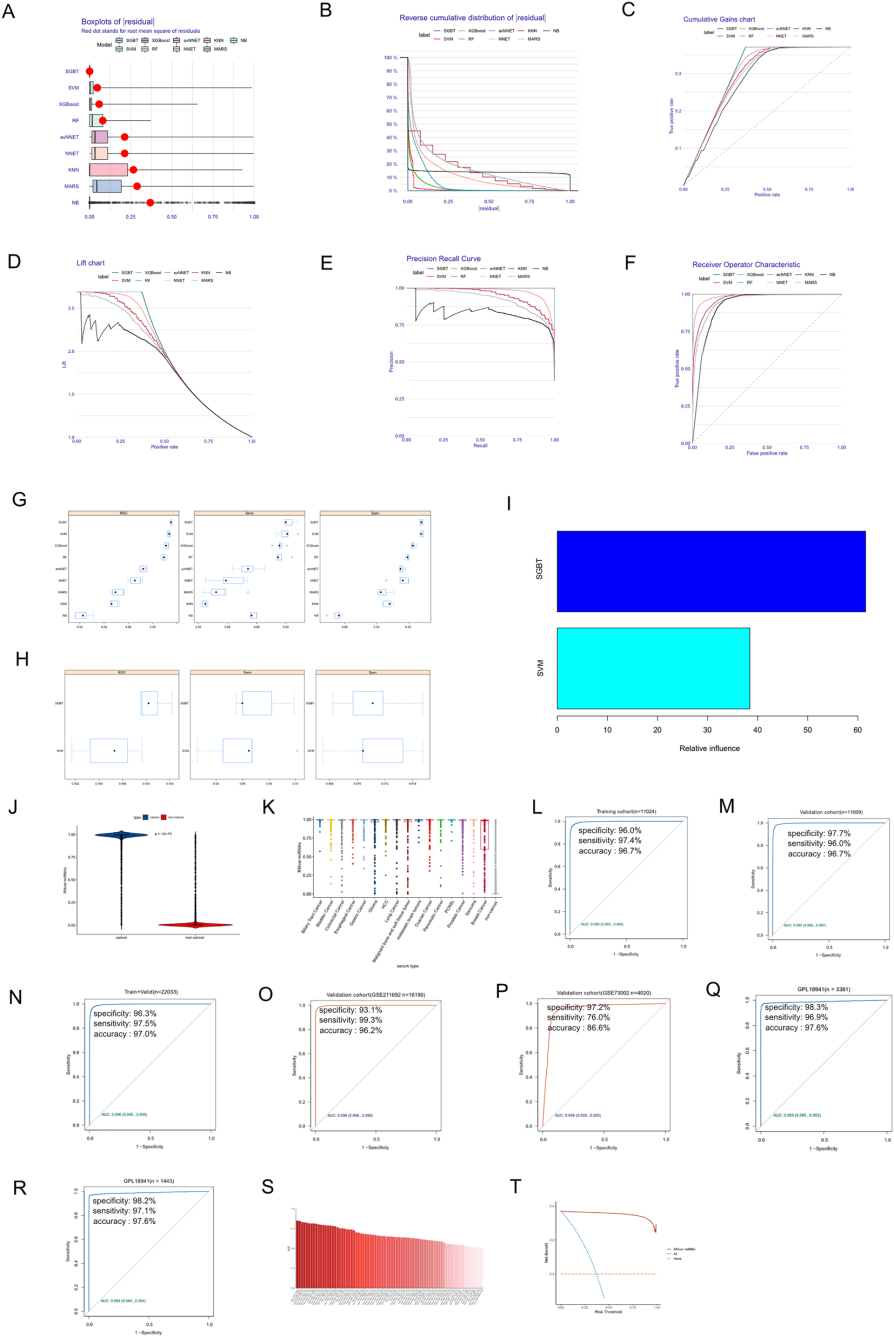
Construction of RMvar-related miRNA signature using machine learning algorithms. (**A**–**F**) Performance of signatures constructed using nine machine learning algorithms on related indices, including residual, cumulative gains, lift chart, precision recall curve, and ROC. (**G**, **H**) AUC, specificity, and sensitivity of miRNA signatures constructed using nine machine learning algorithms in distinguishing cancer and non-cancer control samples. (**I**) Influence of machine learning algorithms (SGBT and SVM) in constructing RMvar-related miRNAs. (**J**, **K**) Differences in the output strength of RMvar-related signature between cancer and non-cancer control samples, as well as different cancer types. (**L**) ROC curve showing the diagnostic performance of RMvar-related signature in distinguishing cancer from non-cancer controls in the training cohort. The AUC, specificity, sensitivity, and accuracy were also calculated. (**M**, **N**) The diagnostic performance of RMvar-related signature as validated in the test cohort and combined cohort using the ROC curve. (**O**, **P**) The diagnostic performance of RMvar-related miRNA signature as validated in the validation cohort using the ROC curve. (**Q**, **R**) The miRNA signature was reconstructed and validated in the combined cohort from the platform of GLP18941. (**S**) AUC of ROC of 79 representative miRNAs in distinguishing cancer and non-cancer control samples. (**T**) The difference in net benefit between RMvar-related miRNAs and all representative miRNAs using the decision curve analysis (DCA) within a wide range of decision threshold probabilities.

Based on the obtained 79 candidate RMvar-related miRNAs, we used the machine learning algorithm to construct a diagnostic signature, the RMvar-related miRNA signature, for cancer detection. First, serum samples (sequencing via the GPL21263 platform; cancer = 8,187, non-cancer control = 13,846) with miRNA expression data from patients with and without cancer were randomly split into two cohorts at a ratio of 1:1 (training and validation cohorts), and nine common machine learning algorithms, namely MARS, RF, NNET, avNNET, SVM with the radial basis function kernel, SGBT, XGBoost, NB, and KNN, were used independently to construct diagnostic signatures based on the training cohort. The hyperparameters for each diagnostic signature were selected according to the best ROC curve (Supplementary Fig. 1). Five machine learning-related indices, including residual, cumulative gains, lift chart, precision recall curve, and ROC, were calculated to compare the predictive power of the nine diagnostic signatures. As shown in Fig. 4A–F, the diagnostic signatures constructed using SGBT and SVM exhibited the best performance in these indices among the above nine diagnostic signatures. The AUC of ROC, sensitivity, and specificity of diagnostic signatures constructed using SGBT and SVM were also highest among the nine diagnostic signatures (Fig. 4G, H). Therefore, we constructed the RMvar-related miRNA signature by combining the SGBT and SVM algorithms using the “caretStack” function of the “caretEnsemble” package, with 100 iterations of bootstrap sampling. The related influences of SGBT and SVM algorithms in constructing the RMvar-related miRNA signature were 63.38% and 36.62%, respectively (Fig. 4I).

The output strengths of these signatures in the cancer groups were significantly lower than those in the non-cancer controls (Fig. 4J). We next investigated the difference in RMvar-related miRNA signature values between each cancer type. As shown in Fig. 4K, in the cancer group, patients with lung cancer had the highest median signature value (1.00). Further, patients with breast cancer had the lowest median signature value (0.64), and a significant difference in signature values was observed between the non-cancer controls and each cancer type (*p* < 0.001). The RMvar-related miRNAs showed high diagnostic power in distinguishing cancer samples from non-cancer controls in the training cohort (Fig. 4L). We then applied the signature to the test cohort. Similar to the training cohort, the RMvar-related miRNAs also showed a high diagnostic performance, with an AUC of 0.996 (95%CI 0.995–0.997), a specificity of 97.7%, and a sensitivity of 96.0%. The diagnostic accuracy was 96.7% (Fig. 4M). We also examined the RMvar-related miRNA signature in the combined training and test cohort. The AUC, specificity, sensitivity, and accuracy demonstrated a satisfactory diagnostic value (Fig. 4N). To examine the predictive power of the miRNA signatures, we analyzed the RMvar-related miRNA signature on two external validation sequencing data GSE211692 and GSE73002. In GSE211692 (from the same sequencing platform as the training cohort), the miRNA signature showed excellent performance in distinguishing cancer samples from non-cancer cases, with an AUC of 0.998 (95% CI 0.998–0.998), specificity of 93.1%, sensitivity of 99.3%, and diagnostic accuracy of 96.2% (Fig. 4O). Examination of the miRNA signatures in the other external validation data (GSE73002) from the sequencing platform GPL18941 indicated slightly lower diagnostic performance with an AUC of 0.948, specificity of 97.2%, sensitivity of 76.0%, and accuracy of 86.6% (Fig. 4P). All cancer cases in miRNA expression data GSE73002 were breast cancer, whose signature value had the largest overlapping parts with non-cancer samples (Fig. 4K). Moreover, the differences in detection technology, detection schemes, and data processing methods across different platforms will affect the presentation of data. These factors may have affected the diagnostic power of RMvar-related miRNA signature in GSE73002. To overcome this, we collected three additional datasets, namely GSE59856, GSE85679, and GES124158, of liver cancer, pancreatic cancer, cholangiocarcinoma, and malignant bone and soft tissue tumor from platform GPL18941 and combined them with GSE73002 to generate new external validation data (named the GPL18941 cohort). Then, we randomly split it into two cohorts at a ratio of 3:1 (training and test cohorts) and constructed diagnostic signatures in the training cohort based on the 79 representative miRNAs and the same signature constructing method. The result showed that the miRNA signature performed well in distinguishing cancer samples from non-cancer controls within the training cohort (Fig. 4Q) and test cohort (Fig. 4R) of GPL18941, with the AUC, specificity, and sensitivity higher than 0.989, 98.2%, and 96.9%, respectively. These results highlight that our optimized machine-learning workflow was effective in constructing a cancer detection tool.

The diagnostic power of each representative miRNA was calculated for the combined training and test cohorts. Our results showed that hsa-miR-320a had the highest diagnostic value, with an AUC of 0.8503, specificity of 80.63%, sensitivity of 79.04%, and accuracy of 79.63%, which was significantly lower than the RMvar-related miRNA signature (Fig. 4S). In the decision curve analyses, the RMvar-related miRNA signature demonstrated superior net benefit within a wide range of decision-making threshold probabilities compared with all the representative miRNAs (Fig. 4T).

### Diagnostic performance of RMvar-related miRNA signature within different conditions and cancer types

**Figure 5:**
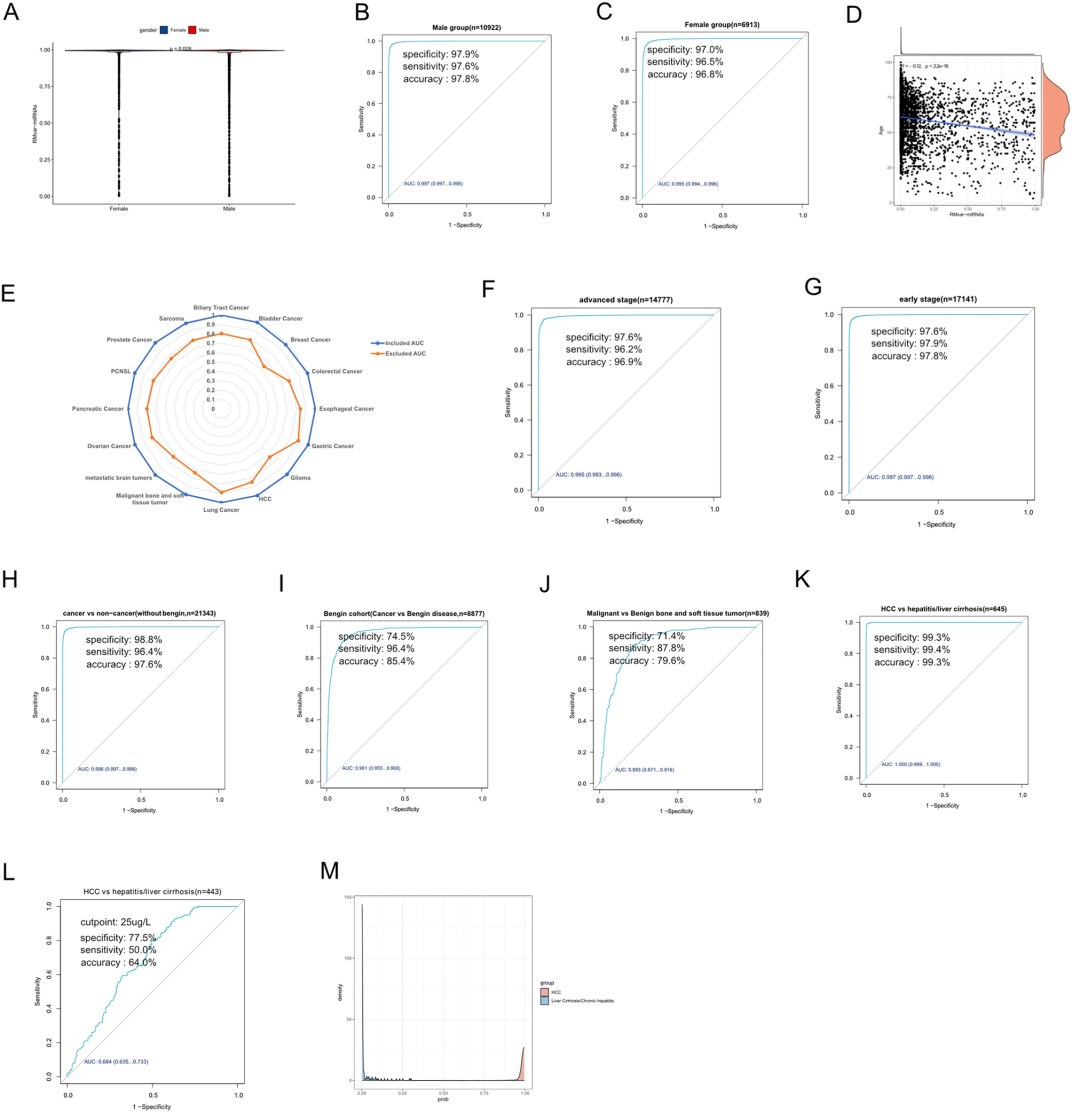
Diagnostic performance of the RMvar-related miRNA signature within different conditions and cancer types. (**A**) Differences in the output strength of the RMvar-related miRNA signature between samples from female and male patients. (**B**, **C**) ROC curve showing the diagnostic performance of the RMvar-related signature in male and female groups. (**D**) Correlation analysis between age and output strength of the RMvar-related miRNA signature in patients with cancer. (**E**) Radar chart summarizing the AUC of the RMvar-related miRNA signature of each cancer type. The blue polyline represents the AUC value for distinguishing each cancer type from non-cancer controls. The yellow polyline represents the AUC value for distinguishing each cancer type from all mixed cancer and non-cancer samples. (**F**–**K**) ROC curve showing the diagnostic performance of the RMvar-related miRNA signature in cohorts corresponding to different conditions. (**L**) ROC curve showing the diagnostic performance of AFP in distinguishing HCC from patients with chronic hepatitis/liver cirrhosis. M: Density of RMvar-related miRNA signature output strength in HCC samples and hepatitis/liver cirrhosis cases.

Our analyses revealed the potent diagnostic value of RMvar-related miRNA signature in cancer detection. We investigated the diagnostic power of RMvar-related miRNA signature in different conditions and certain cancer types. First, we tested the diagnostic performance of RMvar-related miRNA signature classified by patient sex. Our results showed no significant difference in the output strength of the RMvar-related miRNA signature between female (median signature value = 0.99486) and male patients (median signature value = 0.99473) (Fig. 5A), and the AUC of the RMvar-related miRNA signature of both groups performed well, as previously reported (Fig. 5B, C). The correlation analysis showed no significant correlation between patient age and RMvar-related miRNA output strength (cor = − 0.12, Fig. 5D). Therefore, we investigated the ability of RMvar-related miRNAs to distinguish cancer types and combined each cancer type individually with non-cancer control samples. The RMvar-related miRNA signature demonstrated superior discrimination ability (Fig. 5E, blue polyline, Supplementary Table 7). Although the performance of the RMvar-related miRNA signature was slightly lower in distinguishing each cancer type from the mixed samples of all cancer and non-cancer controls, it exhibited a remarkably high sensitivity (Fig. 5E, yellow polyline, Supplementary Table 8). The RMvar-related miRNA signature accurately detected > 87.6% of cancer types (except breast cancer, which had a sensitivity of 64.8%), and the rate of missed diagnosis was low.

Next, we investigated the influence of tumor stage and benign diseases on the diagnostic power of the RMvar-related miRNA signature. Regardless of the advanced or early stages, cancer samples were distinguished accurately from non-cancer controls using the RMvar-related miRNA signature, with AUC values of 0.995 and 0.997, respectively (Fig. 5F, G). Considering the influence of benign diseases on the diagnostic power of RMvar-related miRNA signature, we constructed a cohort that excluded benign diseases and another cohort that only included cancers and benign diseases. As shown in Fig. 5H and I, the RMvar-related miRNA signature demonstrated a high diagnostic power in distinguishing cancer samples from non-cancer controls (excluding benign diseases) or benign diseases, with AUC values of 0.998 and 0.961, respectively, indicating the potent ability of this signature in distinguishing cancers from benign diseases. To confirm this result, we applied this signature to two cohorts involving malignant bone and soft tissue tumors or HCC and their relevant benign diseases. The RMvar-related miRNA signature exhibited a high diagnostic power in distinguishing malignant bone and soft tissue tumors from benign bone and soft tissue tumors, with an AUC of 0.893 (95%CI 0.871–0.916), specificity of 71.4%, sensitivity of 87.8%, and diagnostic accuracy of 79.6% (Fig. 5J). The RMvar-related miRNA signature demonstrated superior performance in distinguishing HCC from hepatitis and liver cirrhosis, with an AUC of 1.000 (95%CI 0.999–1.000), specificity of 99.3%, sensitivity of 99.4%, and diagnostic accuracy of 99.3% (Fig. 5K). These values were superior to those of the traditional biomarker alpha fetoprotein (AFP; AUC = 0.684, specificity = 77.5%, and sensitivity = 50.0%, with a cutpoint of 25 ug/L) (Fig. 5L). The output strength of the RMvar-related miRNA signature in patients with HCC rarely intersected with the value range of patients with chronic hepatitis\liver cirrhosis (Fig. 5M). Our results indicate that the RMvar-related miRNA signature can accurately distinguish cancers, regardless of stage, without a significant interference from related benign diseases.

## Discussion

Genetic variants have been widely used for research in complex human disorders by identifying candidate genes. Numerous genetic variants have been identified to be associated with tumorigenesis^29^. Most studies have focused on nonsynonymous SNVs that alter amino acid sequences, and the SNVs in the non-coding regions have been largely ignored. Recent studies have determined that various miRNAs have variants targeting RNA modifications^18^. These modifications can affect the production, maturation, and metabolism of miRNAs^30,31^. Therefore, the changes in RNA modifications in miRNAs by variants potentially influence their biofunctions and present new features in cancer development. Comprehensively investigating the role of RMvar-related miRNAs provides new strategies to mitigate cancers.

In this study, we collected 504 RMvar-related miRNAs containing nine common RNA modifications. The functional annotation by KEGG and GO enrichment analyses showed that these miRNAs were enriched in viral carcinogenesis, transcriptional misregulation in cancer, pluripotency of stem cells, generation of precursor metabolites and energy, and MAPK signaling pathway NIKNF-kappa B signaling, indicating that these miRNAs may strongly influence the regulation of cell bio-activities and may be closely associated with tumorigenesis.

miRNAs are discharged into a serum by cancer cells through exosomes and reflect the specific molecular signatures of the cancer cells^32^. The high stability of miRNAs ensures their long-term in vitro storage^33^, making them a useful biomarker for cancer detection. Continuous research on the relationship between miRNAs and cancer has identified several miRNAs as cancer biomarkers. For example, the serum miR-1910-3p is an effective diagnostic marker of breast cancer^34^, and high serum miR-1247-3p levels correlate with lung metastasis in patients with HCC^35^. Considering the close relationship between RMvar-related miRNA and tumorigenesis as analyzed in the present study, we propose that Rmvar-related miRNA is a potential biomarker for cancer detection.

We used machine learning algorithms to investigate the diagnostic value of RMvar-related miRNA in cancer detection, as they can process large amounts of data and can be used to construct a comprehensive and precise signature for cancer detection^35^. Of the 504 serum RMvar-related miRNAs, 79 candidate RMvar-related miRNAs, with independent expression, were used to construct a diagnostic signature through the optimized machine learning workflow.

Surprisingly, the RMvar-related miRNA signature constructed using SGBT and SVM was potent in cancer detection, with an AUC of 0.998 (95%CI, 0.998–0.998), a specificity of 93.1%, and a sensitivity of 99.3% in the external validation cohort. The RMvar-related miRNA signature demonstrated a consistently strong performance for different cancer types, wherein > 87.6% of patients with cancer (excluding breast cancer, whose sensitivity was 64.8%) could be identified. Furthermore, the RMvar-related miRNA signature could distinguish cancer from benign diseases and detect early-stage cancers among non-cancer samples. Our results suggest that the RMvar-related miRNA signature is an effective diagnostic tool for cancer detection.

## Conclusion

In this study, we evaluated 504 RMvar-related miRNAs in 43,047 clinical samples and investigated their prognostic value in cancer detection. Using machine learning algorithms, we established RMvar-related miRNA signature and demonstrated their potent performance in distinguishing cancers from non-cancer samples in different clinical conditions. Our study proposed a useful tool for cancer screening.

However, our analyses were based on a public database, which was the primary limitation of this study. In future studies, we intend to use the miRNA PCR array and clinical research to demonstrate the clinical value of RMvar-related miRNA and improve the diagnostic performance of this model.

### Supplementary Information

Supplementary Information 1.

Supplementary Information 2.

Supplementary Information 3.

Supplementary Information 4.

Supplementary Information 5.

Supplementary Information 6.

## Data Availability

The datasets analyzed during the current study are available in the Gene Expression Omnibus (GEO) repository, with accession numbers of GSE106817, GSE112264, GSE113468, GSE113740, GSE122497, GSE124158, GSE137140, GSE139031, GSE164174, GSE73002, GSE211692, GSE59856, GSE85679, and GES124158. The code, preprocessed datasets, and model object for diagnostic signature construction were uploaded as supplementary materials.
