# Supplementary figures and images for: Serum micro-RNAs with mutation-targeted RNA modification: a potent cancer detection tool constructed using an optimized machine learning workflow
Source: Sci Rep. 2024 Apr 19;14:9016. doi: 10.1038/s41598-024-59480-y (PMC11031599; doi:10.1038/s41598-024-59480-y)

# GPL18941(n = 1443)

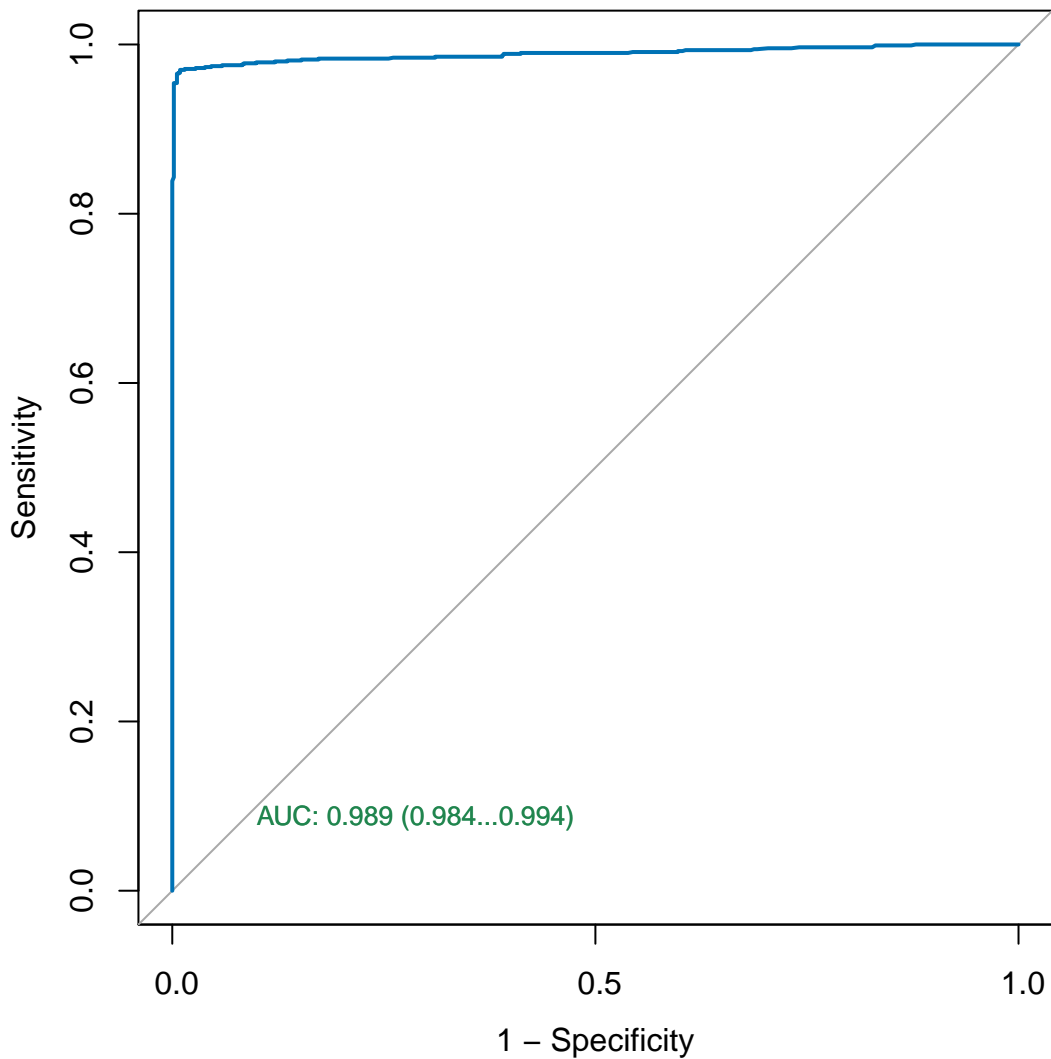

Supplement: Supplementary file 4 — Supplementary Information 4. [file 41598_2024_59480_MOESM4_ESM.zip › 4 GPL18941/4GPL18941/GPL18941(n = 1443).pdf]

# GPL18941(n = 3381)

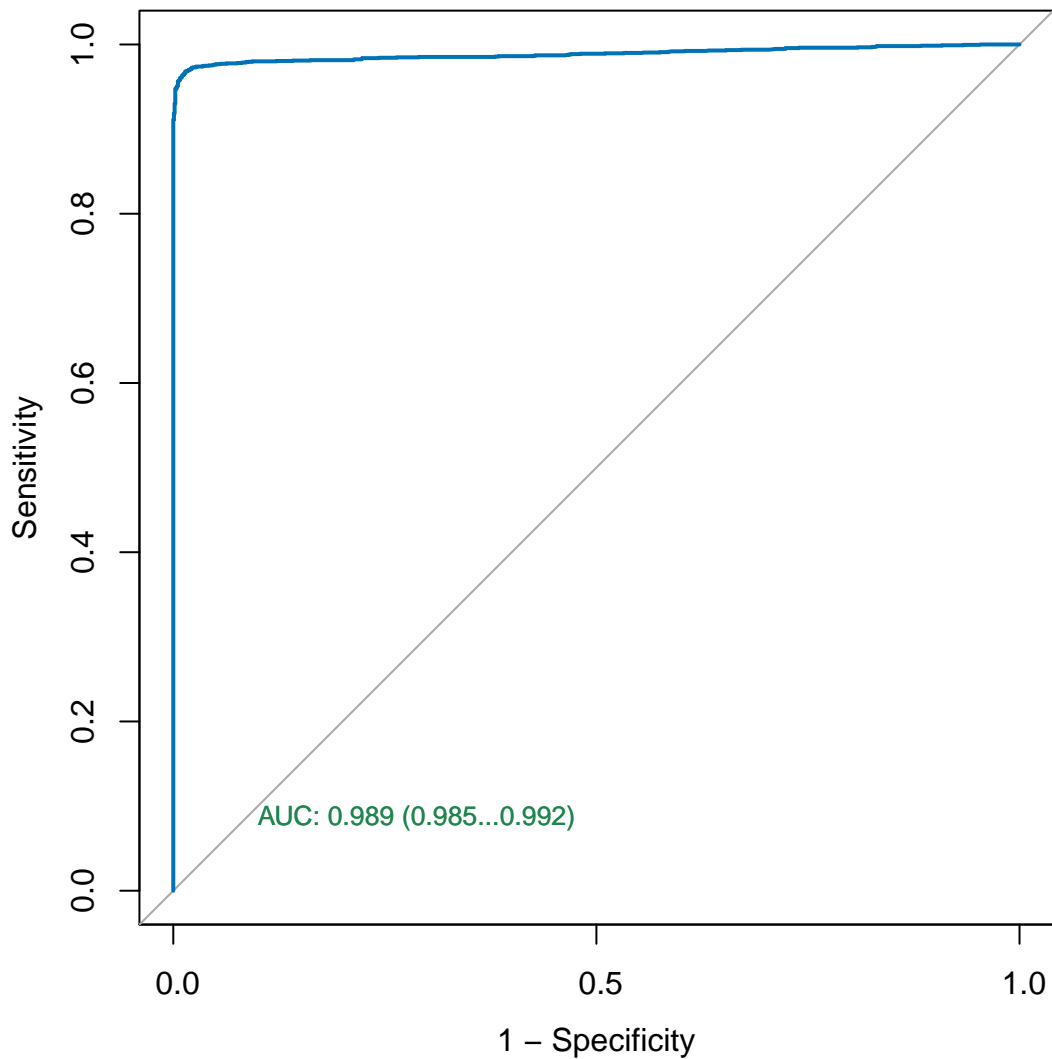

Supplement: Supplementary file 4 — Supplementary Information 4. [file 41598_2024_59480_MOESM4_ESM.zip › 4 GPL18941/4GPL18941/GPL18941(n = 3381).pdf]

# GPL18941(n = 4824)

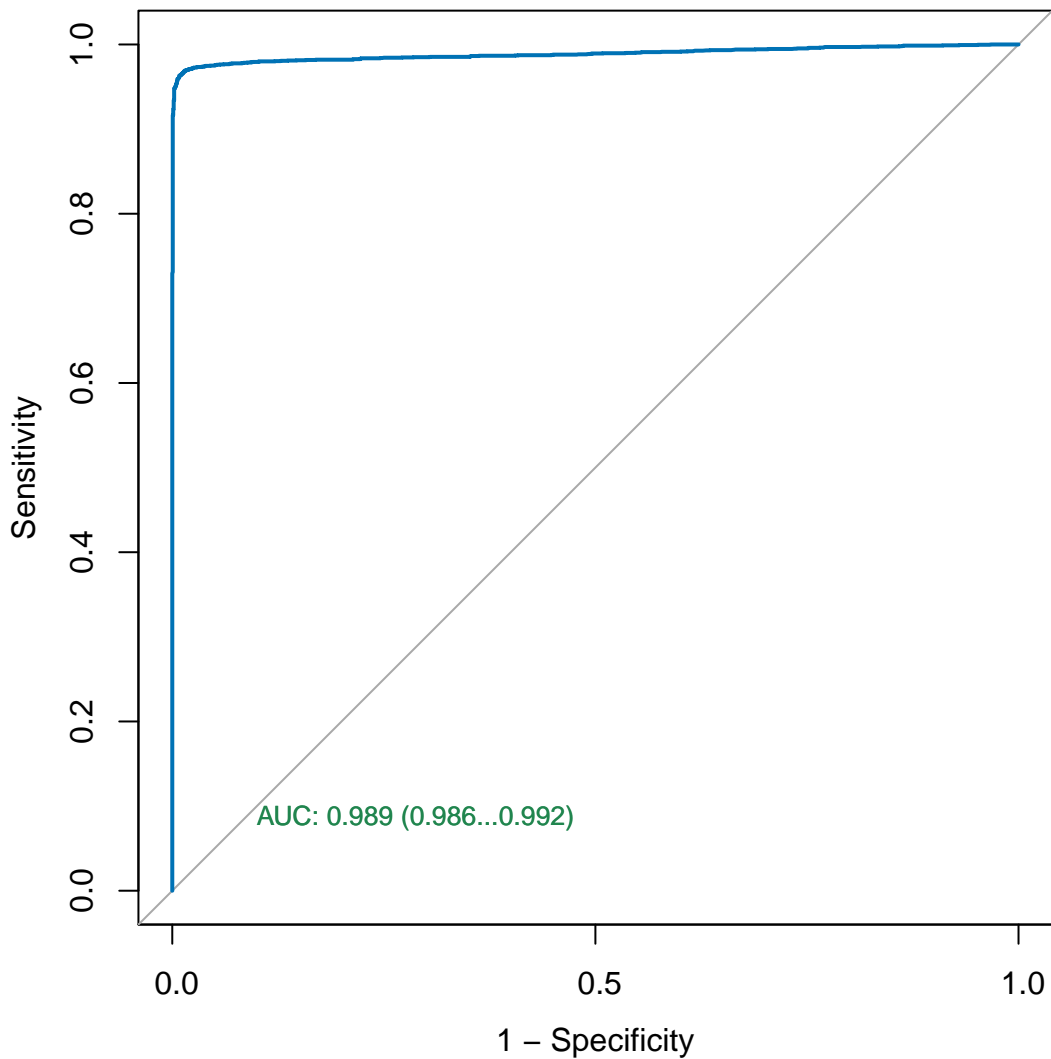

Supplement: Supplementary file 4 — Supplementary Information 4. [file 41598_2024_59480_MOESM4_ESM.zip › 4 GPL18941/4GPL18941/GPL18941(n = 4824).pdf]

**GPL18941(n = 4824)**

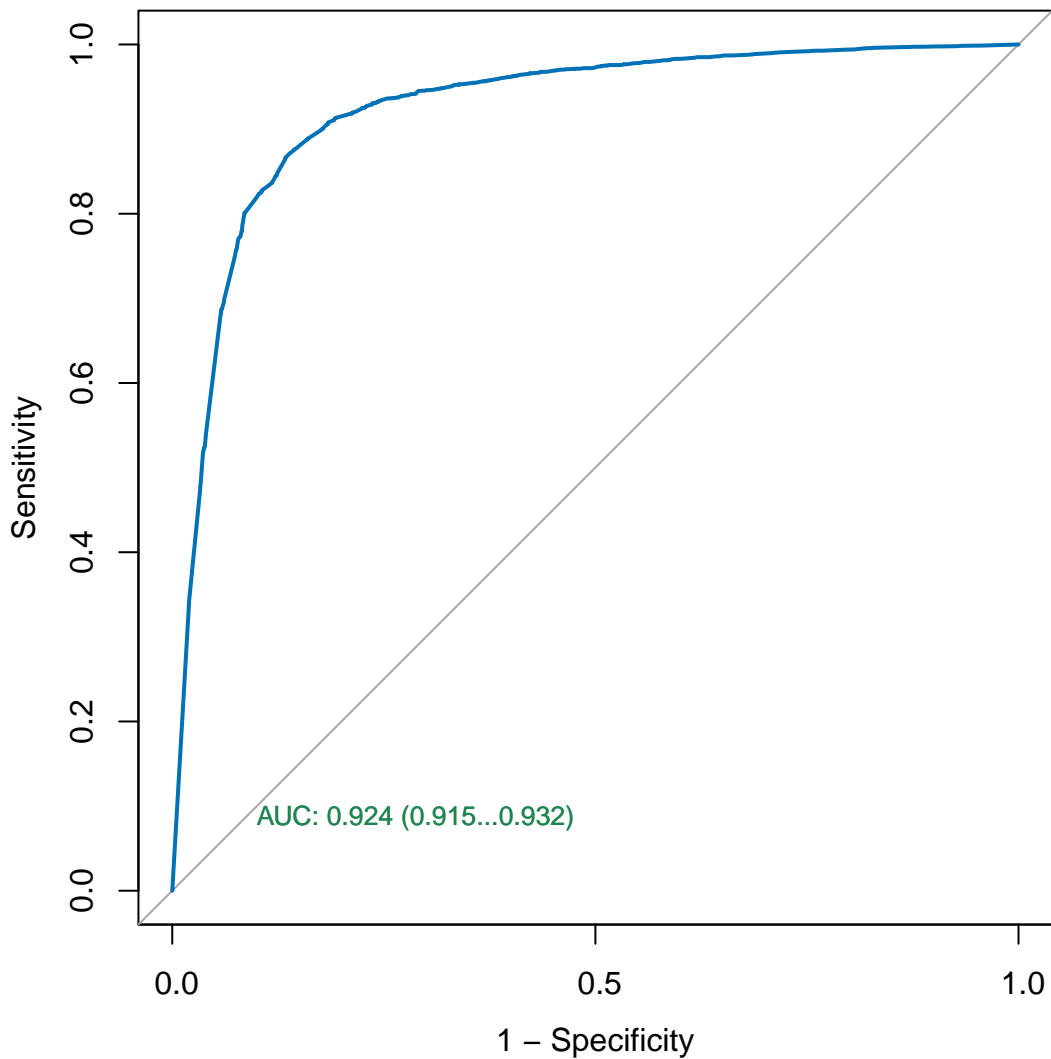

Supplement: Supplementary file 4 — Supplementary Information 4. [file 41598_2024_59480_MOESM4_ESM.zip › 4 GPL18941/4GPL18941/GPL18941.pdf]
