# Supplementary material for: Serum micro-RNAs with mutation-targeted RNA modification: a potent cancer detection tool constructed using an optimized machine learning workflow
Source: Sci Rep. 2024 Apr 19;14:9016. doi: 10.1038/s41598-024-59480-y (PMC11031599; doi:10.1038/s41598-024-59480-y)
Supplement: Supplementary file 5 — Supplementary Information 5. [file 41598_2024_59480_MOESM5_ESM.docx]

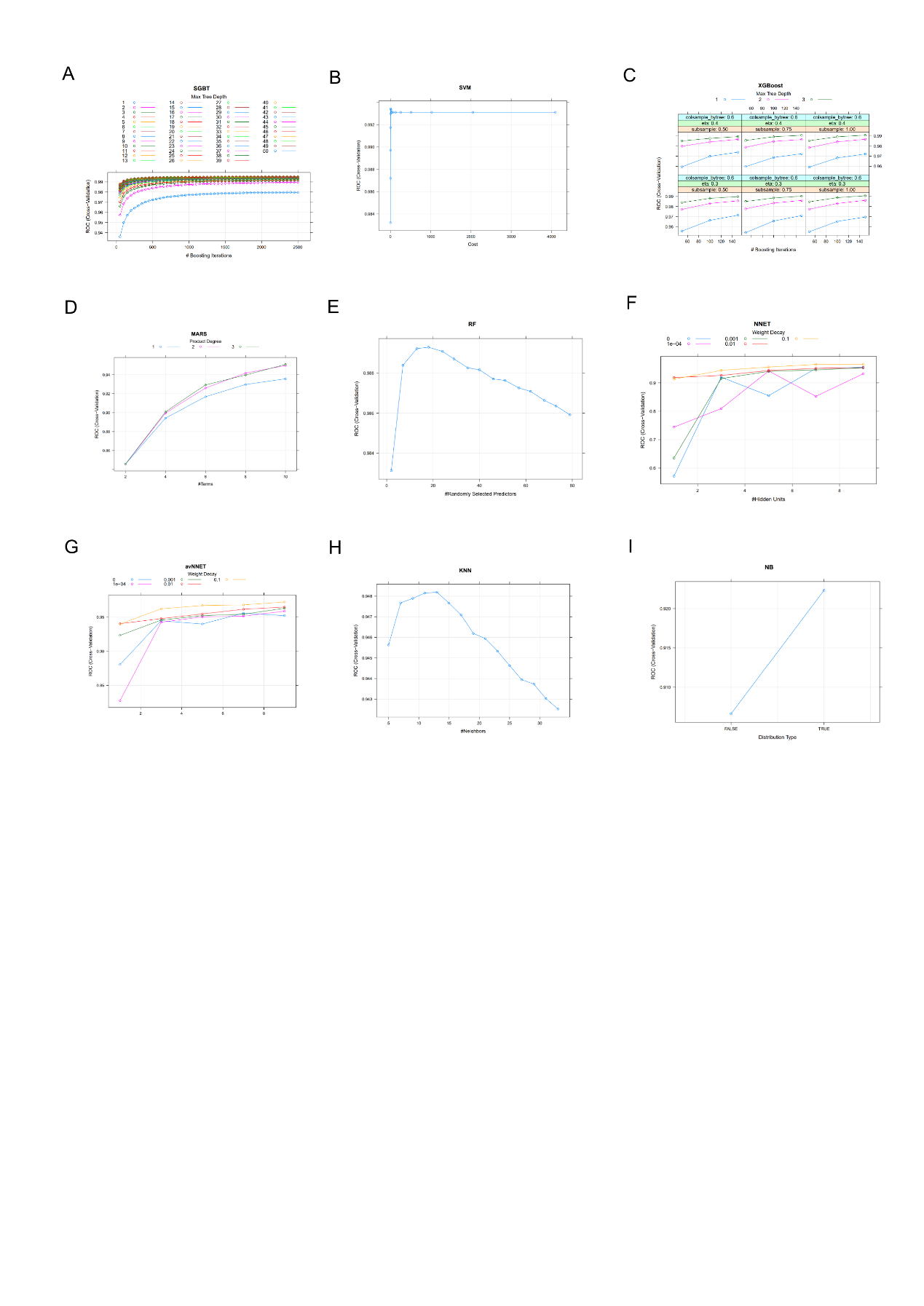


**Supplementary Figure 1. A-I:** Hyperparameters of nine machine learning algorithms chosen according to the best ROC curve. SGBT: Stochastic Gradient Boosting Tree, SVM: Support Vector Machines with radial basis function kernel, XGBoost: eXtreme Gradient Boosting, MARS: Multivariate Adaptive Regression Spline, RF: Random Forest, NNET: Neural Network, avNNET: Model Averaged Neural Network, KNN: k-Nearest Neighbors, and NB: Naive Bayes.
